# Evaluation of NMME temperature and precipitation bias and forecast skill for South Asia

**DOI:** 10.1007/s00382-017-3841-4

**Published:** 2017-08-01

**Authors:** Benjamin A. Cash, Julia V. Manganello, James L. Kinter

**Affiliations:** George Mason University, Center for Ocean-Land-Atmosphere Studies, Fairfax, VA USA

**Keywords:** Monsoon, ENSO, Forecast skill

## Abstract

Systematic error and forecast skill for temperature and precipitation in two regions of Southern Asia are investigated using hindcasts initialized May 1 from the North American Multi-Model Ensemble. We focus on two contiguous but geographically and dynamically diverse regions: the Extended Indian Monsoon Rainfall (70–100E, 10–30 N) and the nearby mountainous area of Pakistan and Afghanistan (60–75E, 23–39 N). Forecast skill is assessed using the Sign test framework, a rigorous statistical method that can be applied to non-Gaussian variables such as precipitation and to different ensemble sizes without introducing bias. We find that models show significant systematic error in both precipitation and temperature for both regions. The multi-model ensemble mean (MMEM) consistently yields the lowest systematic error and the highest forecast skill for both regions and variables. However, we also find that the MMEM consistently provides a statistically significant increase in skill over climatology only in the first month of the forecast. While the MMEM tends to provide higher overall skill than climatology later in the forecast, the differences are not significant at the 95% level. We also find that MMEMs constructed with a relatively small number of ensemble members per model can equal or outperform MMEMs constructed with more members in skill. This suggests some ensemble members either provide no contribution to overall skill or even detract from it.

## Introduction

The North American Multi-Model Ensemble (NMME) was established as a means of exploiting a highly useful and somewhat counter-intuitive property of seasonal forecast models. Multi-model ensemble means (MMEMs), where multiple instances of different models are combined, typically have higher forecast skill than the individual models they are composed of (e.g. Palmer et al. 2004; Jin et al. 2008; Kirtman et al. 2014). This property has resulted in the pursuit of multi-model ensembles (MMEs) as a means of improving intraseasonal to interannual (ISI) prediction capabilities. The success of the MME methodology combined with the straightforward, but not simple, nature of its implementation led to the creation of the National (now North American) Multi-model Ensemble (Kirtman et al. 2014). This initial effort consisted of an extensive hindcast dataset, as well as real-time forecasts, composed of integrations from numerous prominent North American models, including the current US operational seasonal forecast system (Coupled Forecast System version 2; CFSv2). Following the initial success of the NMME (sometimes referred to as Phase-1) a second effort, is ongoing, with updated models and an experimental design more closely in line with the needs of the operational community.

The NMME data represents one of the most extensive archives of seasonal predictions made using active seasonal forecast models currently available (Kirtman et al. 2014), and as such are of tremendous interest and value to both the research and forecasting communities. These represent only some of the most recent studies making use of this extensive and unique data set. Recent studies making use of NMME data to investigate different processes and phenomena include: Shukla and Kinter (2016), which used NMME CFSv2 forecasts to investigate wave heights in the Indian Ocean. Kang and Lee (2016) investigated the predictability of the Arctic Oscillation. Shukla et al. (2016) investigated predictability of the east African monsoon. Villarini et al. (2016) and Manganello et al. (2017) looked at the skill of multi-model forecasting of tropical cyclone activity, while Infanti and Kirtman (2016) considered the diversity of El Niño-Southern Oscillation (ENSO) responses.

Efforts to predict the evolution of the monsoon season date back to at least the late 1800’s (e.g., Blanford 1884). The majority of these efforts have been statistical in approach, with attempts focusing on identifying associations between the monsoon and features such as ENSO, and statistical techniques are still used by the Indian Meteorological Department (IMD) to make the operational monsoon forecast. In more recent years, advances in dynamical modeling have made dynamical prediction of the monsoon feasible (see DelSole et al. 2012; references therein). Analysis of the models used in the ENSEMBLES (Hewitt 2004) project (DelSole et al. 2012; Rajeevan et al. 2012) showed that dynamical models could achieve relatively high correlations with observed Indian summer monsoon rainfall (ISMR) and even exceed the values obtained by regressing on the NINO3 index. Wang et al. (2015) also examined dynamical monsoon prediction, and found that while the ENSEMBLES/CliPass models produced accurate forecasts of the monsoon for some periods, for the most recent decades forecast skill is relatively low. They attributed this to the recent weakening of the ENSO-monsoon relationship; however Cash et al. (2016) demonstrated that the recent ‘weakening’ can be explained by sampling variability, and the actual relationship may be unchanged. On the other hand, it is entirely possible that the recent period is more heavily influenced by climate noise and non-ENSO sea surface temperature (SST) anomalies, making the more recent period more difficult for models to reproduce.

In the current work we expand upon these previous studies by investigating the skill of the NMME in reproducing month-by-month temperature and precipitation anomalies in two different regions of South Asia. We also examine the skill of the MMEM and individual component models for both the full NMME and an NMME constructed with a more limited sample of ensemble members. Section 2 describes the data set and verification methodology chosen, Sect. 3 presents our results, and Sect. 4 summarizes our results and conclusions.

## Data and methods

Measuring progress in forecasting requires a measure of skill and an assessment of whether an observed improvement in skill could be explained by random sampling variability. Standard statistical tests of the difference in mean square error or correlation are not generally valid because these tests assume the quantities being compared are independent, whereas this assumption does not hold if skill is measured relative to observations over the same period (DelSole and Tippet 2014).

Despite these complications, there exist alternative methods that can rigorously assess whether differences in skill could be explained by sampling variability. Perhaps the simplest is the Sign test, in which the hypothesis to be tested is that two forecasts are equally “skillful”. (see DelSole and Tippet 2014 for a discussion of this and other skill comparison tests based on Wilcoxon’s Signed-Rank Test, the Morgan–Granger–Newbold Test, and a permutation test). One expression of this hypothesis is to suppose that forecast A is just as likely to beat forecast B as forecast B is to beat forecast (A) that is, forecast A has a 50% probability of beating forecast (B) if subsequent forecasts are independent, such as for seasonal forecasts separated by a year, then the counts of the number of times forecast A beats forecast B follows the same statistics as that of a fair coin landing on heads after being flipped, namely a Bernoulli process (Table 1).

**Table 1:** NMME models

Model	Hindcast period	Ensemble size	Forecast lead (months)	Native atmos. res.	Native ocean res.	References
NCEP-CFSv1	1982–2009	15	0–8	T62L64	MOM3L40 0.3°Eq	Saha et al. (2006)
NCEP-CFSv2	1982–2010	24	0–9	T126L64	MOM4L40 0.25°Eq	Saha et al. (2014)
GFDL-CM2p1	1982–2010	10	0–11	2 × 2.5°L24	MOM4L50 0.3° Eq	Delworth et al. (2006)
GFDL-CM2p1 aer04	1982–2010	10	0–11	2 × 2.5°L24	MOM4L50 0.3° Eq	
GFDL-CM2p5 FLORB01	1982–2010	12	0–11	C18L32 (50 km)	MOM4L50 0.3° Eq	Vecchi et al. (2014)
GFDL-CM2p5 FLORA06	1982–2010	12	0–11	C18L32 (50 km)	MOM4L50 0.3° Eq	
CMC1-CanCM3	1982–2010	10	0–11	T63L31	CanOM4L40 0.94° Eq	Merryfield et al. (2013)
CMC2-CanCM4	1982–2010	10	0–11	T63L315	CanOM4L40 0.94° Eq	
NCAR-CCSM3 (COLA-RSMAS)	1982–2010	6	0–11	T85L26	POPL42 0.3°Eq	Kirtman and Min (2009)
NCAR-CCSM4 (COLA-RSMAS)	1982–2010	10	0–11	0.9 × 1.25°L26	POPL60 0.25°Eq	Infanti et al. (2016)
NASA-GMAO-062012	1982–2010	11	0–11	1 × 1.25°L72	MOM4L40 0.25°Eq	Vernieres et al. (2012)
NASA-GMAO	1982–2010	11	0–11	1 × 1.25°L72	MOM4L40 0.25°Eq	
IRI-ECHAM4p5-DC	1982–2010	12	0–7	T42L19	MOM3L25 1.5° × 0.5°	DeWitt et al. (2005)
IRI-ECHAM4p5-AC	1982–2010	12	0–7	T42L19	MOM3L25 1.5° × 0.5°	

A key element of the Sign test framework is that it does not make any assumption about the distribution of the forecasts, so it can be applied to non-Gaussian variables like precipitation, and can be applied to any skill measure of individual forecasts, which means it can be used to compare forecasts according to whatever metric is of economic or societal interest. Moreover, the test can compare a single model with a multi-model forecast even if the multi-model mean includes the single model (i.e., the test does not require that the forecasts being compared are composed of separate forecasts). The estimates of relative skill from the Sign test are also not inherently affected by the size of the ensemble used, which is a significant problem in making comparisons between different MME formulations using ranked probability skill score (RPSS) and many other probabilistic measures of forecast skill (Kirtman et al. 2014).

To make the skill comparisons, we first remove the climatological error for each grid point and forecast lead for the season being tested. We analyze the available^1^ hindcasts for the common period 1983–2009. While data are available beyond 2009, the hindcast periods for some models end and others drop out of the NMME entirely. Given this we choose to limit the period considered in order to maximize the consistency and availability of the data analyzed. Integrations are initialized May 1 and run from May through November. To avoid any form of artificial skill the climatological error is calculated in a cross-validated manner, in which the year being tested is not included in the determination of the bias to be removed. In addition, since the CFS-v2 model includes a jump discontinuity in the climatology between 1998 and 1999 (Saha et al. 2014), the climatology for all models is calculated in two separate pieces: 1983–1998 and 1999–2009. All anomalies and skill comparisons for all models are calculated using cross validation for the appropriate period and then combined to produce the final result. Results are validated against the Global Historical Climate Network (GHCN) gridded temperature product (Lawrimore et al. 2011) and the Climate Prediction Center Merged Analysis of Precipitation (CMAP) gridded precipitation product (Xie and Arkin 1997). All data (both models and observations) are distributed and analyzed on a common 1° by 1° grid.

## Results

**Fig. 1:**
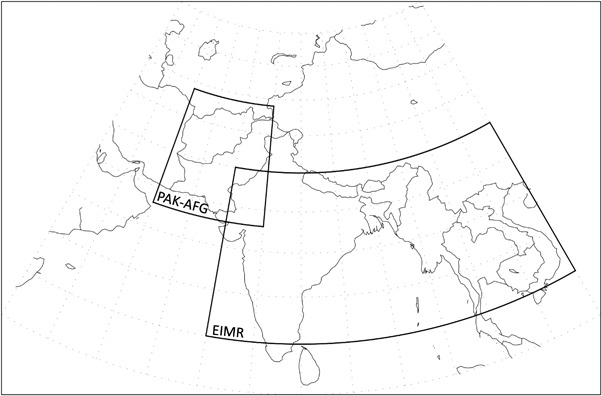
Delineation of the PAK–AFG (60–75E, 23–39 N) and EIMR (70–110E, 10–30 N) regions that are the focus of this study

As described above, our analysis focuses on hindcast skill for temperature and precipitation in two regions of South Asia (Fig. 1) for integrations initialized May 1 and extending through to November. The first is the area that defines the Extended Indian Monsoon Rainfall index (EIMR), which is roughly centered on the Bay of Bengal and covers much of southeast Asia. The EIMR region also encompasses the locations of the rainfall maxima of the South Asian Summer Monsoon. The second region is centered on the nearby mountainous countries of Pakistan and Afghanistan (PAK–AFG) and represents a very different climatic and geographic regime. Focusing on these two regions allows us to test the ability of the models to predict temperature and precipitation through the summer season in two quite different geographic and climatic regions of South Asia.

**Fig. 2:**
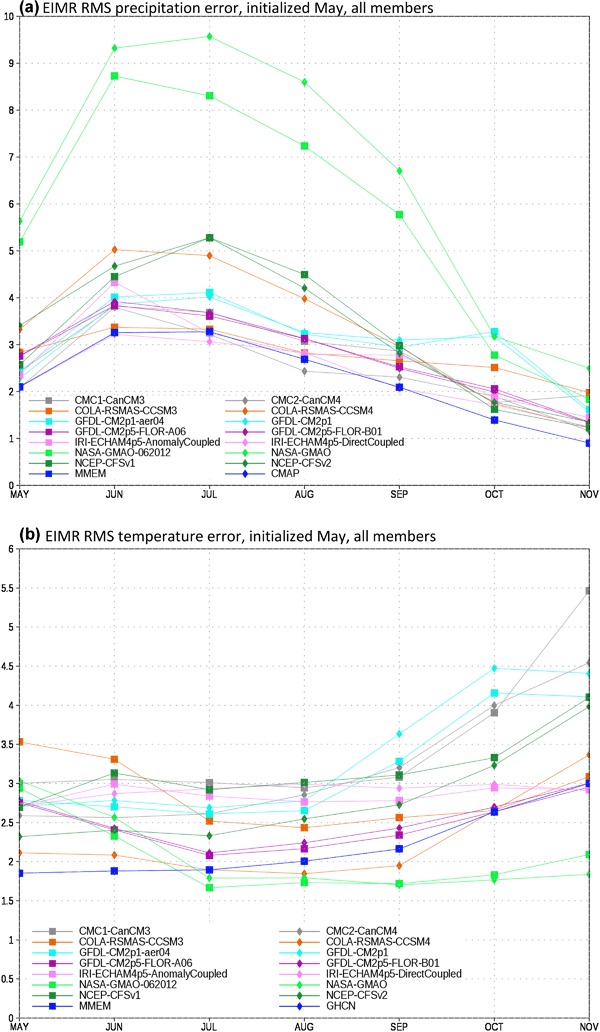
EIMR domain-averaged RMS error for **a** precipitation and **b** temperature for individual models, plus the MMEM. Units are in mm/day and degrees Kelvin, respectively

We first assess the climatological RMS error in precipitation for the EIMR region (Fig. 2a). As part of our investigation into the construction of the MMEM we consider two separate formulations. In the first all available members of the NMME are used. In the second the number of ensemble members is restricted to six for each model, where six is the largest common value. For the CFS models, which use a lagged ensemble, the six members closest in time to the May 1 initial date are chosen. For each model and formulation we calculate the squared error at each grid point, average over the domain, then take the square root to determine the RMSE for the domain. We choose to take the domain average of the squared error, as opposed to the squared error of the domain average, in order avoid the situation where compensating errors within the chosen domains lead to artificially low error values.

From Fig. 2a it is apparent that there is a large degree of spread in the model errors for this metric and region. The two NASA contributions are clear outliers relative to the other NMME models from May to September, which can be traced to significant overestimation of precipitation over the Tibetan Plateau (see Singh et al. 2017). The COLA-CCSM4, NCEP-CFSv1, and NCEP-CVFv2 models have the next highest error values and the rest of the models fall roughly together in a group. The MMEM generally has the lowest error at all leads. Error magnitudes decrease sharply towards the end of the simulation, denoting the end of the rainy season in models and observations alike. The mean error is essentially identical whether all members or only six are used (not shown). Mean error is thus a robust property of each model and apparently insensitive to variations in the initial conditions.

The climatological error for temperature in the EIMR region (Fig. 2b) shows some sharp contrasts to the precipitation errors. In particular, the two versions of the NASA model have some of the lowest systematic errors relative to the other models. The relative position of the different models is also not constant throughout the season. For example, while the COLA-CCSM4 model has a relatively large error at the beginning of the run, by the end its errors are among the lowest. Consistent with the precipitation results, we find there is little sensitivity to the choice of ensemble members. We also find that the MMEM is again among the best models in terms of systematic error. Although there are individual months where one model or another outperforms the MMEM, no models do so systematically.

**Fig. 3:**
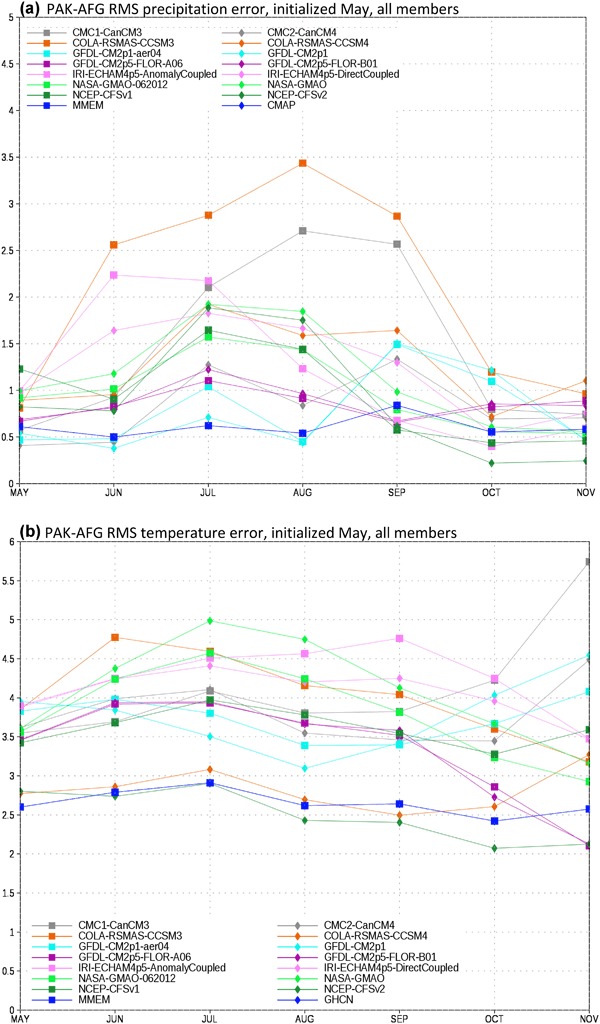
PAK-AFG domain-averaged RMS error for **a** precipitation and **b** temperature for individual models, plus the MMEM. Units are in mm/day and degrees Kelvin, respectively

Turning our attention to precipitation in the PAK-AFG region (Fig. 3a), we again find that certain models are clear outliers. However, the roster of models is now different, with COLA-CCSM4 and CMC2-CanCM4 now standing out as having the largest errors. The NASA models’ errors are now commensurate with the rest of the NMME, and various models outperform the MMEM for the period of August through November. This reshuffling of relative model fidelity with variable and region highlights the difficulties in identifying a single set of “best” models, as the relative positions can change dramatically.

Consistent with what we found for EIMR, the PAK-AFG temperature error grouping (Fig. 3b) is quite different from what we found for precipitation (Fig. 3a). There is a much less pronounced seasonality to the errors, and no models are obvious outliers. Interestingly, we find that for this region and variable, the NCEP-CFSv2 and CCSM4 are closely competitive with the MMEM, marking the only time the MMEM is not a clear improvement over the individual member models in terms of RMS error for the formulations of the MMEM tested in this work.

**Fig. 4:**
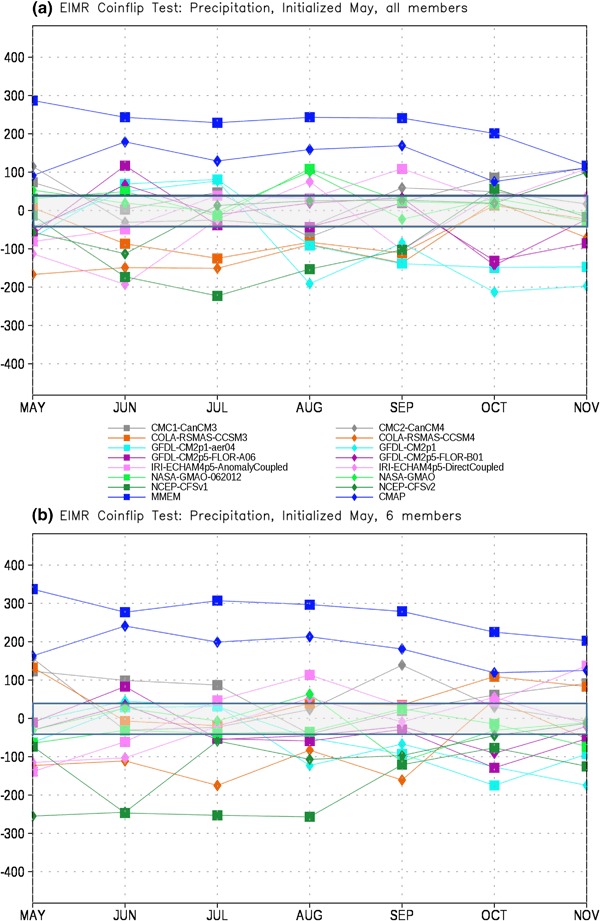
Total wins minus losses for each model for EIMR precipitation for **a** all members retained and **b** six members retained. *Grey bar* denotes values that are not significantly different from the expected result of a fair coin at the 95% level

**Fig. 5:**
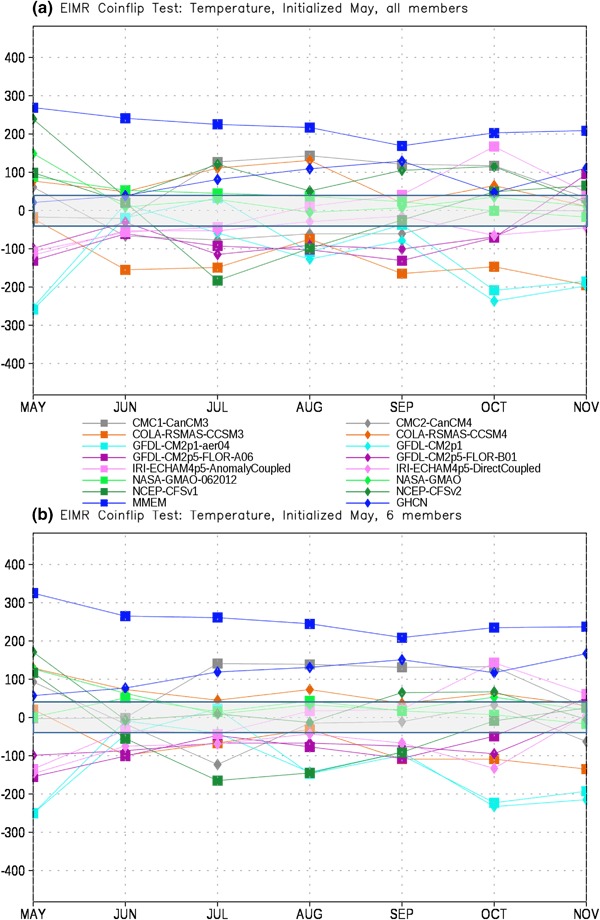
Total wins minus losses for each model for EIMR temperature for **a** all members retained and **b** six members retained. *Grey bar* denotes values that are not significantly different from the expected result of a fair coin at the 95% level

**Fig. 6:**
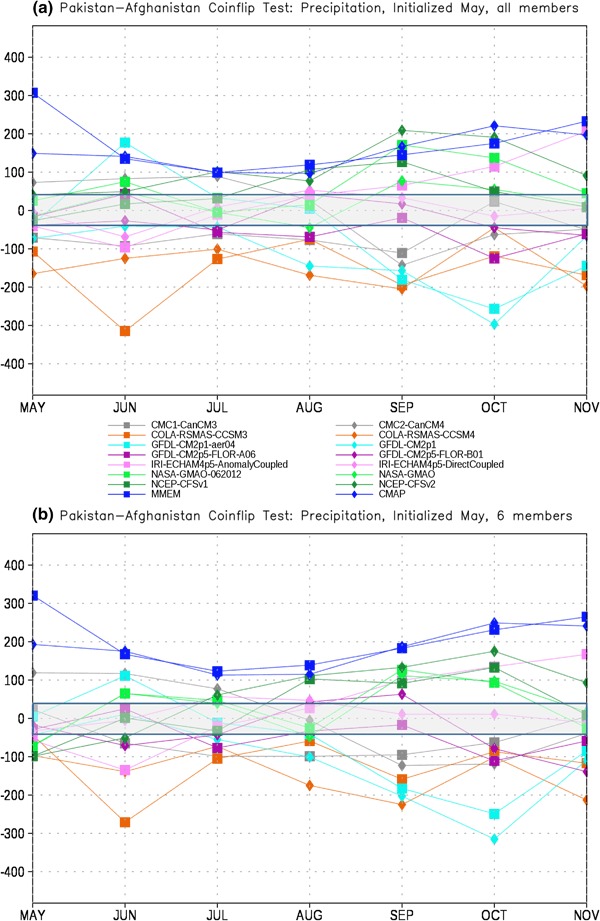
Total wins minus losses for each model for PAK-AFG precipitation for **a** all members retained and **b** six members retained. *Grey bar* denotes values that are not significantly different from the expected result of a fair coin at the 95% level

**Fig. 7:**
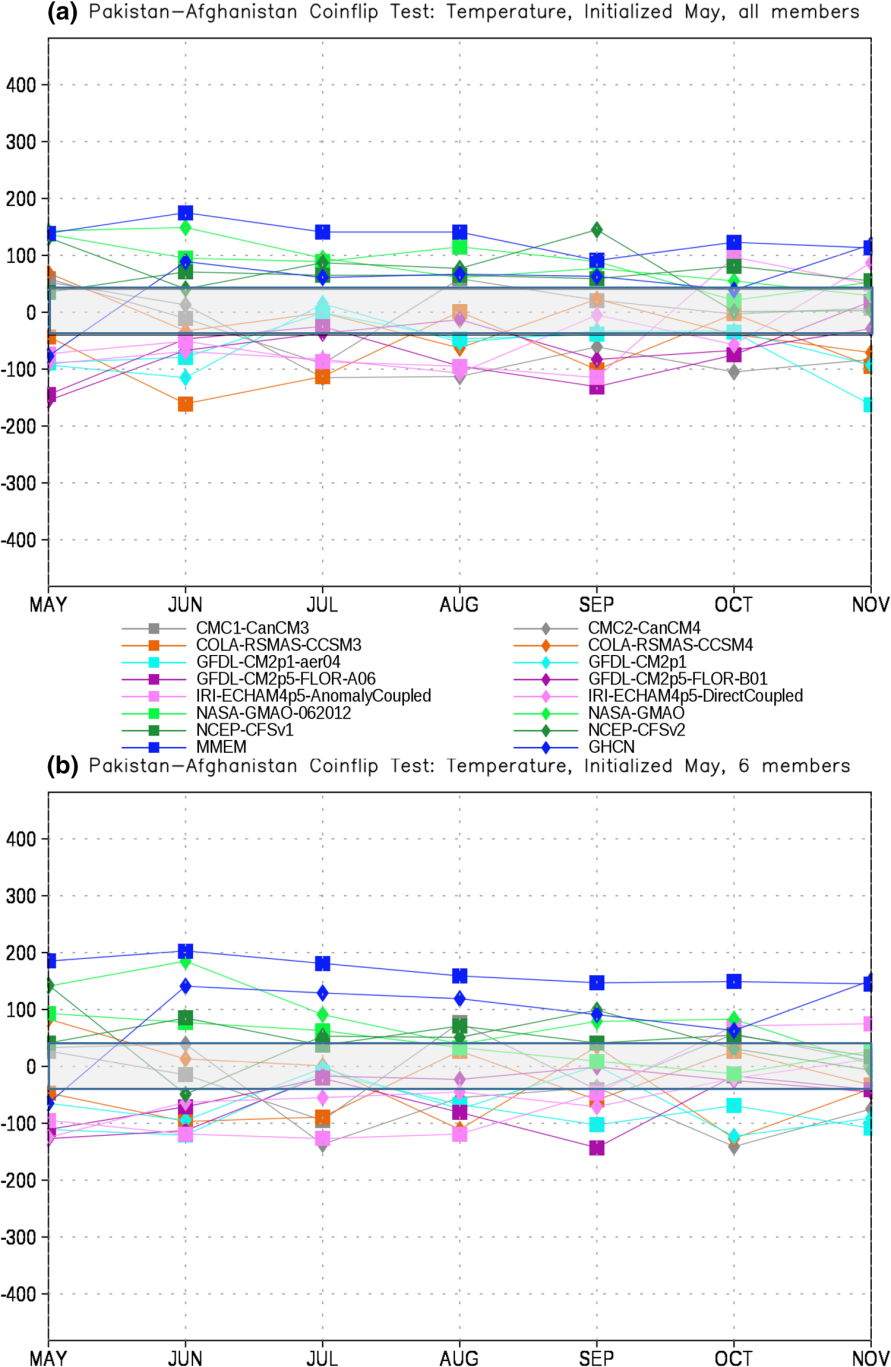
Total wins minus losses for each model for PAK-AFG temperature for **a** all members retained and **b** six members retained. *Grey bar* denotes values that are not significantly different from the expected result of a fair coin at the 95% level

In order to assess the forecast skill for the different members of the NMME, as described in Sect. 2 we first test each model pair-wise against every other model for each month. The number of times a given model is the closest model in the pair to the observations is tallied. This tally is then summed over all model pairs to produce the total number of ‘wins’ and ‘losses’ for each model against all other models. The total number of losses are subtracted from the wins for each lead, producing the curves in Figs. 4, 5, 6 and 7. It is important to recall that the coin flip test compares pairs of models and thus assesses relative, rather than absolute, skill. It can thus be difficult to interpret the utility of the skill differences identified by the test. Out of two extremely poor forecasts, for example, one will inevitably be somewhat closer to the observations than the other. The more accurate model would be considered the winner in the comparison, regardless of the absolute magnitude of the error.

**Fig. 8:**
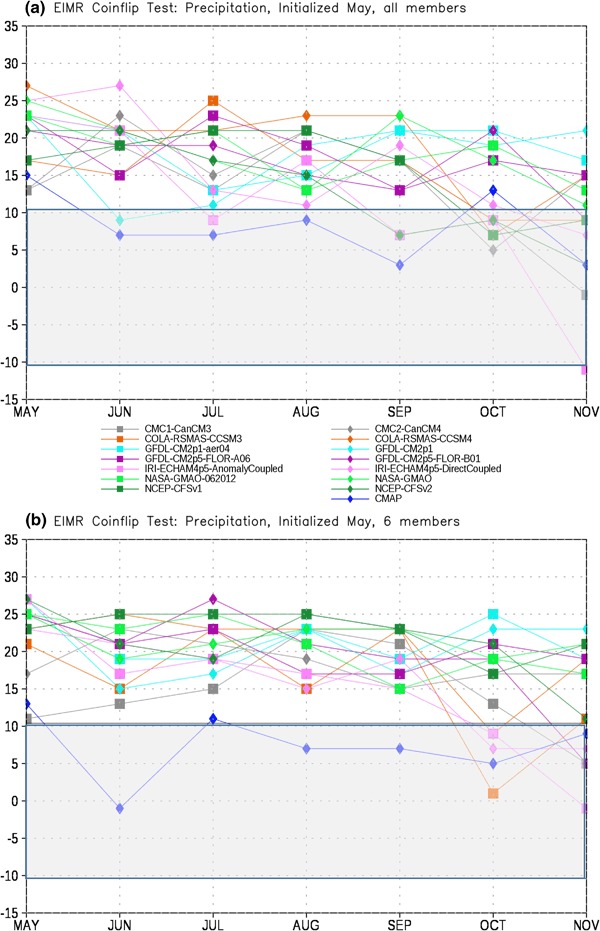
Total wins minus losses for the MMEM against each other model for EIMR precipitation for **a** all members retained and **b** six members retained. *Grey bar* denotes values that are not significantly different from the expected result of a fair coin at the 95% level

**Fig. 9:**
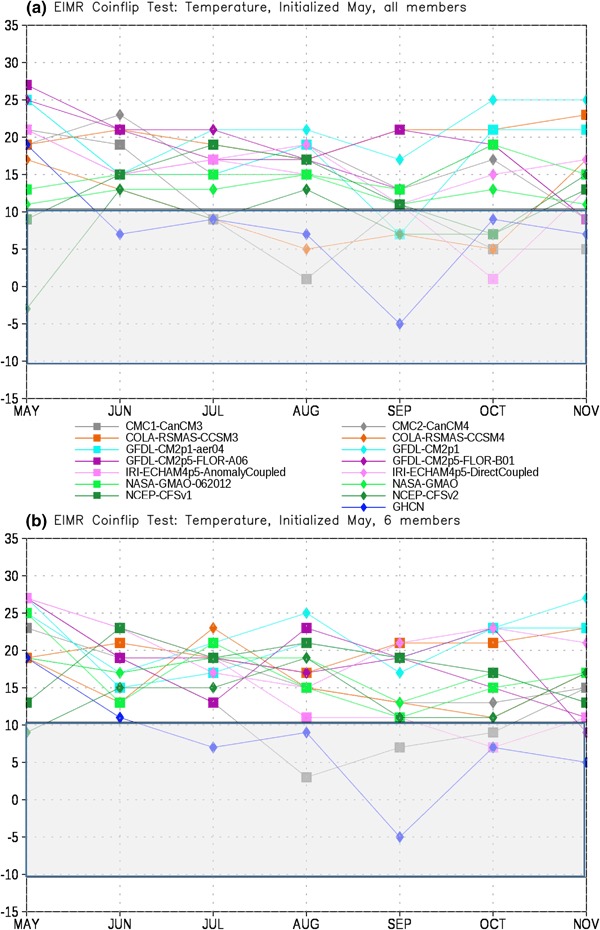
Total wins minus losses for the MMEM against each other model for EIMR temperature for **a** all members retained and **b** six members retained. *Grey bar* denotes values that are not significantly different from the expected result of a fair coin at the 95% level

**Fig. 10:**
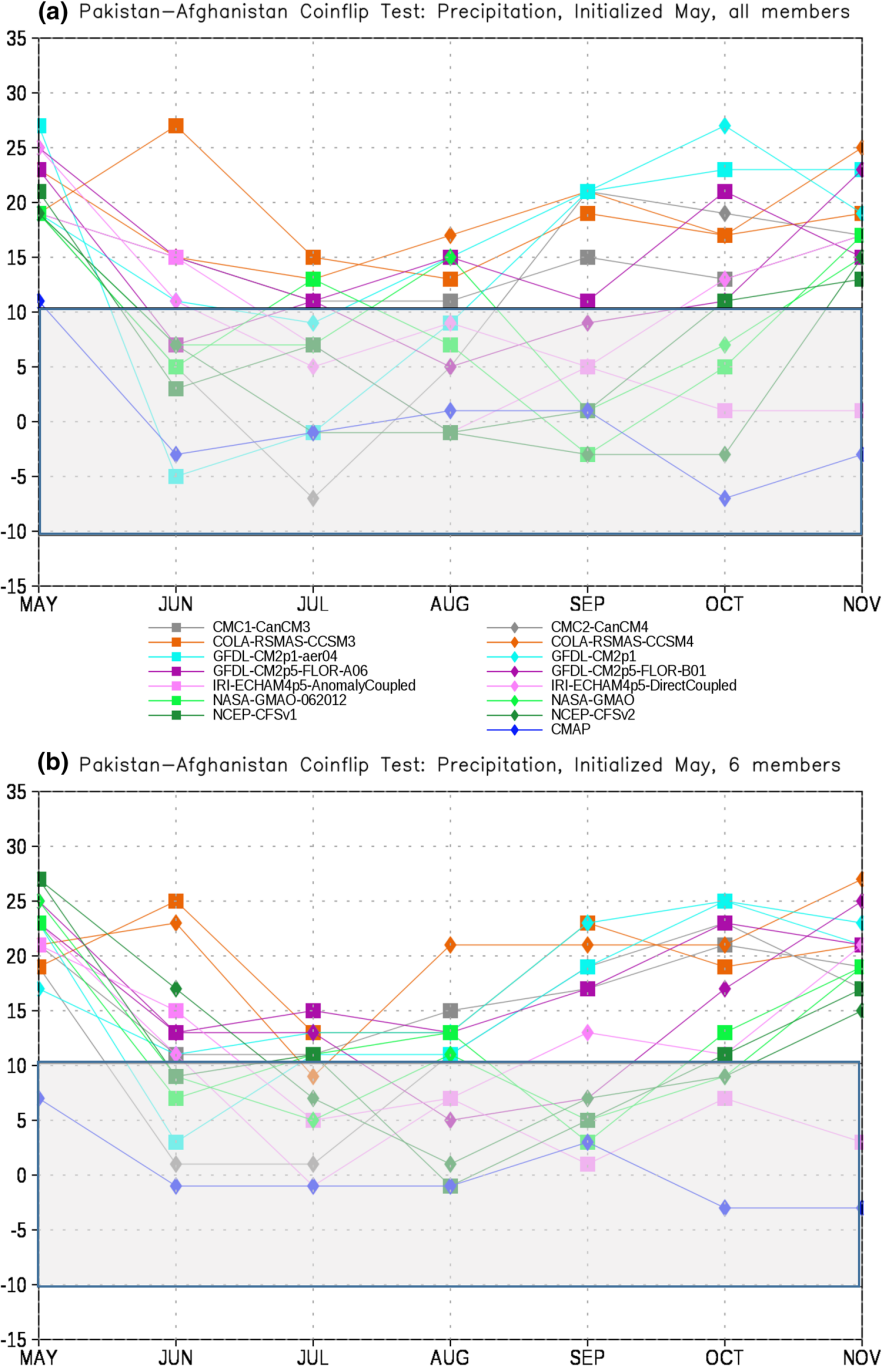
Total wins minus losses for the MMEM against each other model for PAK-AFG precipitation for **a** all members retained and **b** six members retained. *Grey bar* denotes values that are not significantly different from the expected result of a fair coin at the 95% level

**Fig. 11:**
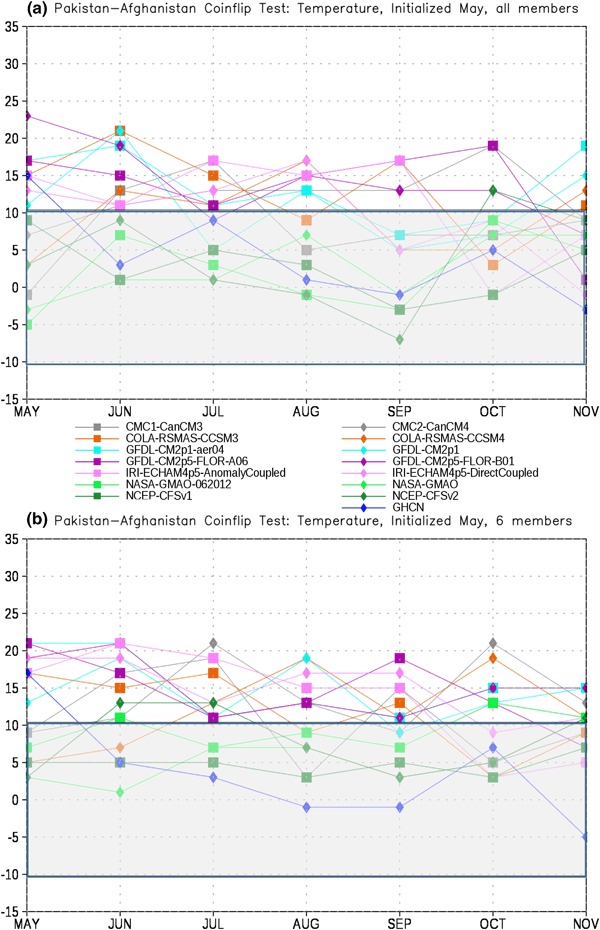
Total wins minus losses for the MMEM against each other model for PAK-AFG temperature for **a** all members retained and **b** six members retained. *Grey bar* denotes values that are not significantly different from the expected result of a fair coin at the 95% level

To place the relative skill of the forecasts in context we also include the observed climatology as an additional ‘member’ of the ensemble. The skill of the various models relative to climatology is taken as a measure of the added value of running the models over simply relying on climatology. As noted in Sect. 2, statistical significance is based on the expected difference in heads and tails in the flip of a fair coin. The grey bar in Figs. 4, 5, 6, 7, 8, 9, 10 and 11denotes values that are not significantly different from equal skill at the 95% level.

For EIMR precipitation, in the case where all members are used (Fig. 4a) the MMEM is closest to the observed anomaly more often than any other member, including climatology. Climatology (CMAP) has the second highest number of successes, exceeding that of any individual member of the NMME. The rest of the models cluster around the expected value for a coin flip, with no notable outliers. The results are generally insensitive to reducing the size of the ensemble to six members each (Fig. 4b), although there is some minor reshuffling of position. Somewhat surprisingly, the relative position of NCEP-CFSv2 is not significantly altered by the reduction in ensemble size, despite having by far the largest ensemble. This potentially reflects a relatively minimal contribution by the older members of the lagged ensemble to the overall skill. Of particular interest is the fact that while the NASA model (both versions) has the largest mean state error in this region and variable (see Fig. 2a) they are in the upper tier of hindcast skill. They are significantly better relative to the flip of a fair coin than the other NMME models for several of the months considered, highlighting the fact that mean state error and forecast skill are not always related.

As with precipitation the MMEM provides the best forecast of EIMR temperature more often than any other option considered (Fig. 5). However, unlike precipitation we now see several models that outperform climatology (GHCN), even at relatively long leads. The CFS models, the CanCM, and the anomaly coupled IRI-ECHAM model all outperform climatology at various leads, particularly for October. The performance of the models relative to climatology does show some sensitivity to the choice of ensemble size, with climatology more consistently outperforming the dynamical models when ensemble size is limited to 6 (Fig. 5b), although the anomaly coupled IRI-ECHAM model is still superior in October.

The PAK-AFG precipitation test shows a very different character from EIMR rainfall (Fig. 6). Here the separation between MMEM, CMAP and the individual members is much less prominent, and there are leads where individual models (GFDL-AERO4, NCEP-CFSv2) exceed the number of successes of both. The total successes for MMEM and CMAP overlap at most leads, unlike for EIMR, indicating that the models are relatively less skillful for the PAK–AFG region. This is perhaps not unexpected, given the complex and mountainous terrain of the region, and the relatively low resolution of the simulations, and is consistent with the lower correlation with observed seasonal rainfall in this region for two different formulations of the MMEM (see Singh et al. 2017, Fig. 7). Reducing the number of ensemble members decreases the relative position of CFSv2, eliminating the leads where CFSv2 is an improvement over the MMEM (Fig. 6b). As with precipitation, the separation between the MMEM and the individual models in PAK–AFG for temperature (Fig. 7) is not as prominent as for EIMR (Fig. 5). However, the MMEM remains the best option available, except for one model at one lead. The MMEM also exceeds the number of successes of GHCN at all leads, indicating that it is generally the more accurate option. Taken together the results of Figs. 4, 5, 6 and 7 show that the MMEM is typically closer to the observations than all individual members of the NMME, and outperforms climatology as well. However, the comparison between total wins and losses does not allow for direct assessment of the skill of any individual approach, such as the MMEM, against any specific individual member. In particular, the relative number of wins against climatology cannot be determined from this metric-only the overall performance can be assessed.

To address the head-to-head performance of the MMEM in Figs. 8, 9, 10 and 11 we show the number of wins-losses for the MMEM against the individual models that comprise it, as well as climatology. Figure 8 shows the comparison of the MMEM against all other options for EIMR precipitation. We find that in the first month of the forecast we can reject the hypotheses that the MMEM is of equal skill for all other options, including climatology. For June–November the MMEM continues to score the greatest number of success in comparison to all models (with one or two exceptions for individual models and leads). However, the hypothesis of equal skill cannot be rejected in the comparison with climatology at the 95% level. Interestingly, reducing the ensemble to six members each (Fig. 8b) improves the performance of the MMEM relative to the rest of the NMME (note the general upward shift in Fig. 8b relative to Fig. 8a), while leaving the performance relative to climatology generally unchanged. This behavior is generally repeated for the comparison with temperature (Fig. 9), where we now find significant improvement in skill over climatology for May and June for the six-member case relative to the full NMME. For most leads the MMEM hovers near significance, with the notable exception of September.

As we might expect from Figs. 6 and 7, the performance of the MMEM over the PAK-AFG region is substantially worse than for EIMR. For precipitation the full ensemble case (Fig. 10a) shows improvement over climatology that barely exceeds the 95% level in the first month and falls below zero at most of the longer leads. In contrast to EIMR there is no improvement as the ensemble is reduced (Fig. 10b), and in fact the first month difference with climatology is no longer significant. This general pattern is repeated for temperature (Fig. 11) where the first month is significant, but after that there is little to no benefit to using the model over climatology.

**Fig. 12:**
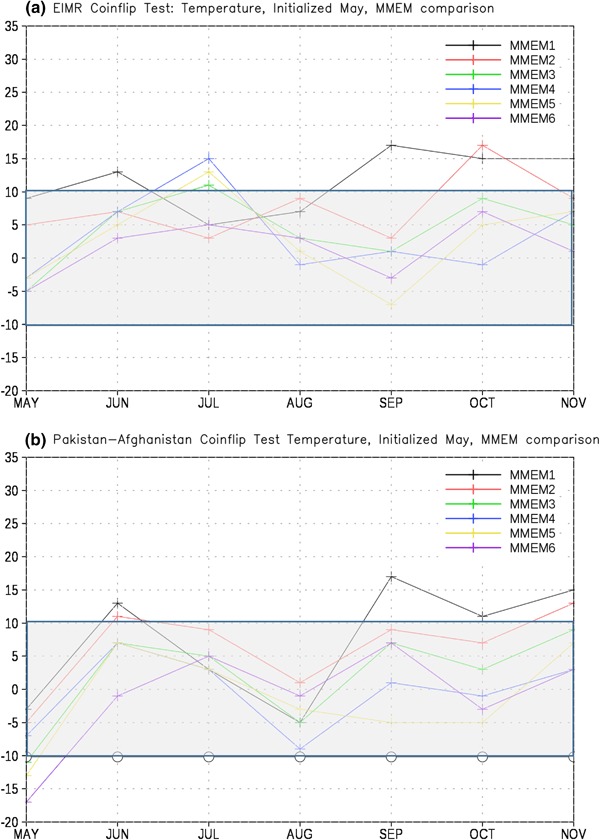
Total wins minus losses for the all-member MMEM against other MMEM formulations for **a** EIMR and **b** PAK-AFG precipitation. *MMEM{1,2,3,4,5,6}* denotes number of members retained. *Grey bar* denotes values that are not significantly different from the expected result of a fair coin at the 95% level

**Fig. 13:**
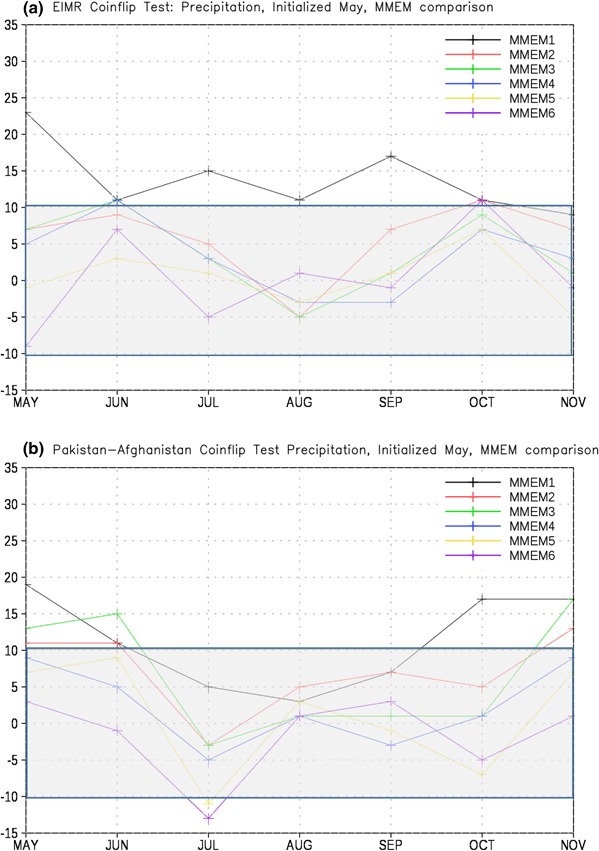
Total wins minus losses for the all-member MMEM against other MMEM formulations for **a** EIMR and **b** PAK-AFG temperature. *MMEM{1,2,3,4,5,6}* denotes number of members retained. *Grey bar* denotes values that are not significantly different from the expected result of a fair coin at the 95% level

To further investigate the skill of the MMEM as a function of the number of retained members, we created five additional MMEMs in which 1–5 ensemble members were retained from each model. As with the six-member formulation described previously, the members closest to the May 1 start date are retained for the lagged ensembles. The five-member formulation is thus a subset of the six-member formulation, and so on. These MMEMs were then tested against each other for our two regions and variables (Figs. 12, 13). For EIMR precipitation we find that the full member ensemble outperforms the one member formulation (MMEM1) by a statistically significant margin for most months. However, we find no statistically significant difference in skill even with the MMEM2 formulation. For PAK-AFG precipitation (Fig. 12b) we find that the full member ensemble is significantly better than MMEM1–3 for May, June, and November, but in general skill is statistically equal for the remaining months. The one notable exception to that finding is July, where the MMEM6 formulation again represents an improvement over the full ensemble.

For EIMR temperature (Fig. 13a) we find that the full member ensemble generally outperforms the smaller ensembles, as shown by positive values for most months and most comparisons. However, the number of months and ensemble formulations where the improvement is statistically significant is relatively small, and primarily limited to comparisons with the MMEM1 and MMEM2 formulations. The relative improvement of the full ensemble is even more limited for the PAK-AFG region (Fig. 13b), where we find the MMEM6 formulation is a statistically significant improvement in the first month.

## Summary and conclusions

We have investigated the month-by-month systematic error and forecast skill of the NMME hindcasts for 1983–2009 for temperature and precipitation over the Extended Indian Monsoon Rainfall (70–100E, 10–30 N) and Pakistan–Afghanistan (60–75E, 23–39 N) regions of Southern Asia for forecasts initialized May 1. We find that there is a wide variety in systematic error for both regions and variables, and that error in one region or variable is a poor indicator of performance outside of that region and variable. Ensemble mean model error is generally unaffected by the choice of the number of ensemble members, indicating it is a robust property of each model.

When we rigorously assess model forecast skill using the coin flip test, we find that the association between systematic error and forecast skill is not particularly strong after the mean bias is removed. In one striking example, the NASA models were among the most skilled at forecasting precipitation for the EIMR region, despite having the highest systematic errors for the region. We also found significant region to region and variable to variable differences in skill, highlighting the difficulty in identifying a single ‘best’ model. Reducing the number of ensemble members to 6 leads to some reshuffling of the relative position of the models, but differences are not particularly dramatic.

Of the forecast products, the MMEM stands out clearly as having the highest forecast skill. The number of times where the MMEM is more accurate than any individual model generally exceeds the number that would be expected from equal skill. The MMEM is also generally more accurate than simply using climatology for the region, although the difference in number of successes is often not significantly different from what would be expected from equal skill. The performance of the NMME is clearly dependent on the region and variable considered, with better performance for temperature over precipitation (as one might expect) and better performance over the larger EIMR in comparison to the mountainous PAK-AFG region (as one might also expect). Interestingly, a MMEM constructed using only six members from each model was roughly as skillful as a MMEM using all available members for these variables and regions, and arguably slightly more so. Given that the MMEM generally exceeds the skill of any individual model when compared to climatology, this result suggests that a significantly smaller overall investment in terms of total model runs would yield similar overall skill. It also suggests that at least some of the model ensembles are being generated in such a fashion as to reduce their overall skill.

The fact that the MMEM appears to provide little benefit over climatology is at first glance a rather discouraging result, particularly in light of previous studies showing reasonable skill for dynamical models in predicting the monsoon. However, it should be noted that in attempting to forecast monthly, rather than seasonal, variations in temperature and precipitation we have set a particularly challenging task for the dynamical models. In that light, the fact that the MMEM significantly outperforms climatology for EIMR temperature for May and June can be taken as an encouraging sign. However, the relatively minimal improvements represented by the models over climatology in this metric and the large systematic errors represents a clear and pressing challenge to the dynamical modeling community.

## Footnotes

1 http://iridl.ldeo.columbia.edu/SOURCES/.Models/.NMME/.
